# Gamma Attenuation Features of White Cement Mortars Reinforced by Micro/Nano Bi_2_O_3_ Particles

**DOI:** 10.3390/ma16041580

**Published:** 2023-02-14

**Authors:** Mona M. Gouda, Ahmed M. El-Khatib, Mahmoud I. Abbas, Shoaa Mofleh Al-Balawi, Mahmoud T. Alabsy

**Affiliations:** Physics Department, Faculty of Science, Alexandria University, Alexandria 21511, Egypt

**Keywords:** white cement, bismuth oxide, attenuation coefficients, half value layer, effective atomic number, exposure buildup factor

## Abstract

This study aims to explore the radiation protection properties of white mortars based on white cement as a binder and Bi_2_O_3_ micro and nanoparticles in proportions of 15 and 30% by weight as replacement sand. The average particle size of micro- and nano-Bi_2_O_3_ was measured using a transmission electron microscope (TEM). The cross-sectional morphology and distribution of Bi_2_O3 within the samples can be obtained by scanning electron microscopy (SEM), showing that nanoscale Bi_2_O_3_ particles have a more homogeneous distribution within the samples than microscale Bi_2_O_3_ particles. The shielding parameters of the proposed mortars were measured using the HPGe detector at various γ-ray energies emitted by standard radioactive point sources ^241^Am, ^133^Ba, ^60^Co, ^137^Cs, and ^152^Eu. The experimental values of the prepared mortars’ mass attenuation coefficients (MAC) match well with those determined theoretically from the XCOM database. Other shielding parameters, including half value layer (HVL), tenth value layer (TVL), mean free path (MFP), effective electron density (N_eff_), effective atomic number (Z_eff_), equivalent atomic number (Z_eq_), and exposure buildup factor (EBF), were also determined at different photon energies to provide more shielding information about the penetration of gamma radiation into the selected mortars. The obtained results indicated that the sample containing 30% by weight of nano Bi_2_O_3_ has the largest attenuation coefficient value. Furthermore, the results show that the sample with a high concentration of Bi_2_O_3_ has the highest equivalent atomic numbers and the lowest HVL, TVL, MFP, and EBF values. Finally, it can be concluded that Bi_2_O_3_ nanoparticles have higher efficiency and protection compared to microparticles, especially at lower gamma-ray energies.

## 1. Introduction

Due to the current rapid advancement of technology and the necessity for ionizing radiation in many areas, including nuclear power plants, medicine, research facilities, and industry, gamma and X-ray utilization has taken on a high concern. It requires protecting the environment, people, patients, workers, and the public against its risks [1,2]. Lead and concrete are well-known materials for radiation protection, with concrete being one of the most popular materials used in radiation therapy facilities and nuclear reactors to protect against radiation leakage from radioactive sources [3,4]. As for lead, although it is used as a radiation-shielding material, its toxicity is a significant environmental threat, and the harm it causes to the human body is serious. Thus, there is an urgent need to find sustainable radiation shielding material to protect humans and the environment from the destructive impact of radiation [5,6]. Bismuth oxide Bi_2_O_3_ was selected in this study due to its higher atomic number and nontoxicity; furthermore, it is considered a high-density material suitable for radiation shielding and has been applied to various materials such as glass [7,8], rubber [9,10], and building materials [11,12,13].

Recently, it became clear that science had taken a huge leap forward in the field of nanotechnology, as many researchers have focused on merging it with different materials such as cement and other inexpensive materials to improve the quality and production of the mixture. Plangpleng et al. [14] developed flexible radiation shielding based on NR using BaSO_4_ nanocomposites. Nanofillers were present in concentrations at 0, 10, 20, 30, and 50 phr and the results indicated that the increased concentrations of BaSO_4_ nanoparticles in NR increased the density, hardness, tensile modulus, tensile strength, and elongation at the break of natural rubber, and the radiation shielding properties were improved. El-Khatib et al. [15] studied the effects of particle size and percentage of weight of CdO particles on gamma radiation shielding properties of CdO/HDPE composites. The composites filled with nano-CdO have greater mass attenuation coefficients compared to that filled with micro-CdO at the same weight fraction. That is attributed to the homogenous distribution of nano CdO particles within HDPE matrix with high electron density, which results in higher interaction probability between incident photons and CdO NPs in nanocomposites compared to micro composites. A relative increase rate of about 16.73% is obtained with nano-CdO content of 40wt% at 59.53 keV gamma attenuation coefficients of nano cadmium oxide/high-density polyethylene composites. In another study, Tekina et al. [16] investigated the effects of WO_3_ and Bi_2_O_3_ additions on the radiation shielding capabilities of hematite-serpentine concrete (HSC). In this study, MCNPX (version 2.6.0), a general-purpose Monte Carlo code, was used to determine the mass attenuation coefficient for the HSC concrete mixed with WO_3_ and Bi_2_O_3_ at the micro- and nanoscales for this purpose. The results showed that adding nanoparticles to HSC enhanced its radiation shielding properties, with applications in both civil engineering structures and the potential to be used as a building material for nuclear plants. Additionally, the results of many studies showed that adding different percentages of nanoparticles may eliminate the porosity in concrete, in addition to binding cement particles to each other, which enhances the structure and efficiency of concrete due to its small size, which is usually less than 100 nanometers [16,17,18,19].

The present study suggested using micro- and nano-Bi_2_O_3_ powders to make mortar using white cement and sand. Therefore, mortars differ from concrete. Mortar, which plays a significant role in the overall performance, is a construction paste used to bond masonry units to each other and to fill gaps between them. Mortar is characterized by its low cost, high strength, and durability, in addition to being easy to repair [20,21,22].

The article structure is organized as follows: Section 2 describes materials and methods, and it presents information on materials, morphology tests, and radiation shielding parameters. Additionally, it contains the experimental arrangement measured at the Radiation Physics Laboratory, Physics Department, Faculty of Science, Alexandria University, Alexandria, Egypt. Section 3 contains the results and discussion, showing the Bi_2_O_3_ nanoparticles have higher attenuation compared to microparticles. Conclusions are presented in Section 4.

## 2. Materials and Methods

### 2.1. Materials

This work is based on the use of white cement, which was purchased from Amreyah Cement (Borg El Arab, Egypt), and fine sand. Additionally, two types of bismuth (III) oxide particles were used: Bi_2_O_3_ microparticles, which are 99.9% high-purity powder obtained from Loba Chemie, India, as well as Bi_2_O_3_ nanoparticles, which were chemically prepared by the company “Nanotech” (Cairo, Egypt).

To give an accurate indication of the chemical composition in the samples, the chemical analyses were measured using the energy-dispersive X-ray spectroscopy (EDX) technique (JSM-IT200 Series) in the Faculty of Science, Alexandria University, Egypt. The chemical composition of the fine sand and white cement used in this study is listed in Table 1.

### 2.2. Preparation of Specimens

The preparation of the mortar mixtures in this study was carried out with a fixed water-to-cement ratio of 0.5. Five mortar mixture groups, which are coded as CS, CS-mBi_2_O_3_ 15%, CS-mBi_2_O_3_ 30%, CS-nBi_2_O_3_ 15%, and CS-nBi_2_O_3_ 30%, were prepared according to the mixing mass ratios given in Table 2. A CS mortar mixture was prepared as a reference mixture. All white cement mortar samples were poured into cylindrical plastic molds where the radius of the cylinder is 30 mm and the height is 5 mm. The mixed samples were dried for one week at room temperature (23 ± 1 °C). Using water as the immersion medium, Archimedes’ technique was used to determine the average density (g/cm^3^) of the mortar samples.

### 2.3. Morphology Test

**Figure 1:**
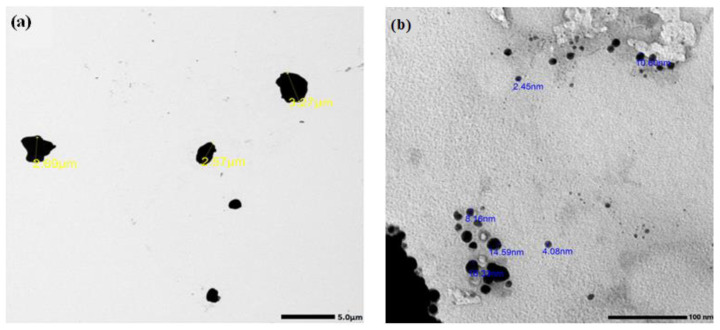
TEM images of (**a**) Bi_2_O_3_ microparticles and (**b**) Bi_2_O_3_ nanoparticles.

**Figure 2:**
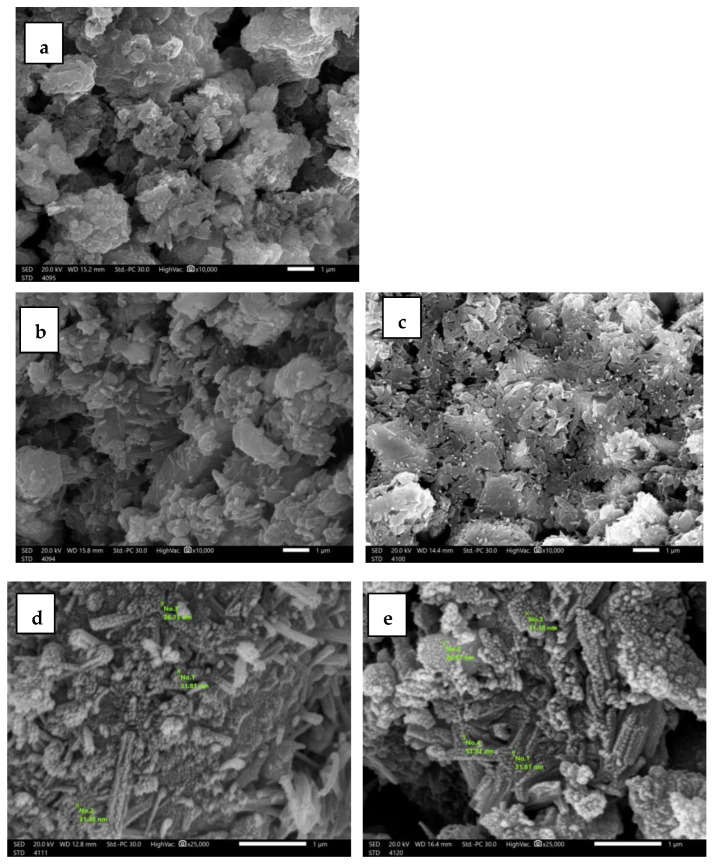
SEM images of the mortar prepared samples of (**a**) CS, (**b**) CS-mBi_2_O_3_ 15%, (**c**) CS-mBi_2_O_3_ 30%, (**d**) CS-nBi_2_O_3_ 15%, and (**e**) CS-nBi_2_O_3_ 30%.

The average particle size of micro and nano Bi_2_O_3_ was measured using a transmission electron microscope (TEM) (JEM-2100F, JEOL, Tokyo, Japan) at 200 kV. The average size of the microparticles is 3 µm and that of the nanoparticles is 8 nm, as shown in Figure 1. The cross-sectional morphology and distribution of Bi_2_O_3_ within the samples can be obtained by scanning electron microscopy (SEM) (JEOL-JFC-1100E). Figure 2a–e shows SEM images of mortar-prepared samples of CS, CS-mBi_2_O_3_ 15%, CS-mBi_2_O_3_ 30%, CS-nBi_2_O_3_ 15% and CS-nBi_2_O_3_ 30%. Figure 2d,e shows that nano Bi_2_O_3_ particles have a more homogeneous distribution within the samples than micro Bi_2_O_3_ particles, which is due to the fact that nanoparticles have a very large surface area-to-volume ratio when compared to the same material in bulk.

### 2.4. Radiation Shielding Properties

The ability of any material to be effective in shielding from gamma rays is determined by studying various important parameters such as linear attenuation coefficient (LAC), mass attenuation coefficient (MAC), half-value layer (HVL), tenth value layer (TVL), mean free path (MFP), and exposure build-up factor (EBF). The shielding parameters were measured using the gamma rays from standard radioactive sources ^241^Am (59.53 keV), ^133^Ba (80.99, 356.01 keV), ^152^Eu (121.78, 244.69, 778.90, 964.13, 1408.01 keV), ^137^Cs (661.66 keV), and ^60^Co (1173.23, 1332.50 keV). These point sources were purchased from The Physikalisch-Technische Bundesanstalt (PTB) (Braunschweig, Berlin, Germany). The certificates give the sources’ activities and their uncertainties for (PTB) sources are listed in Table 3.

**Figure 3:**
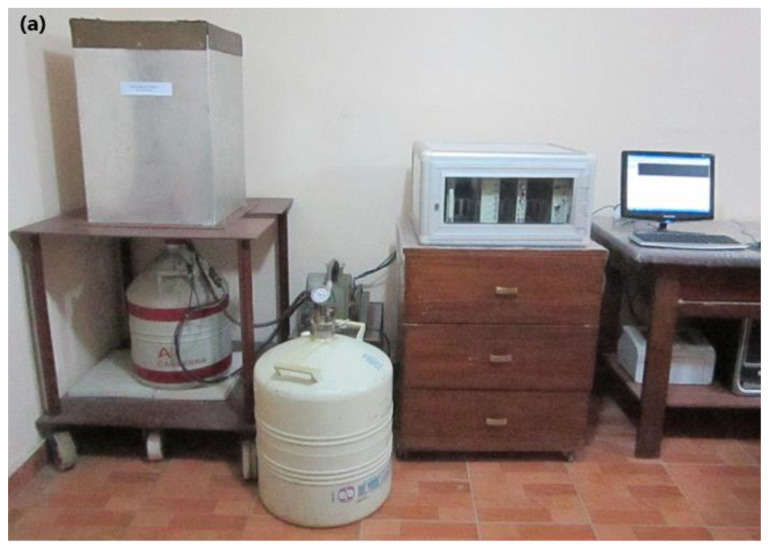

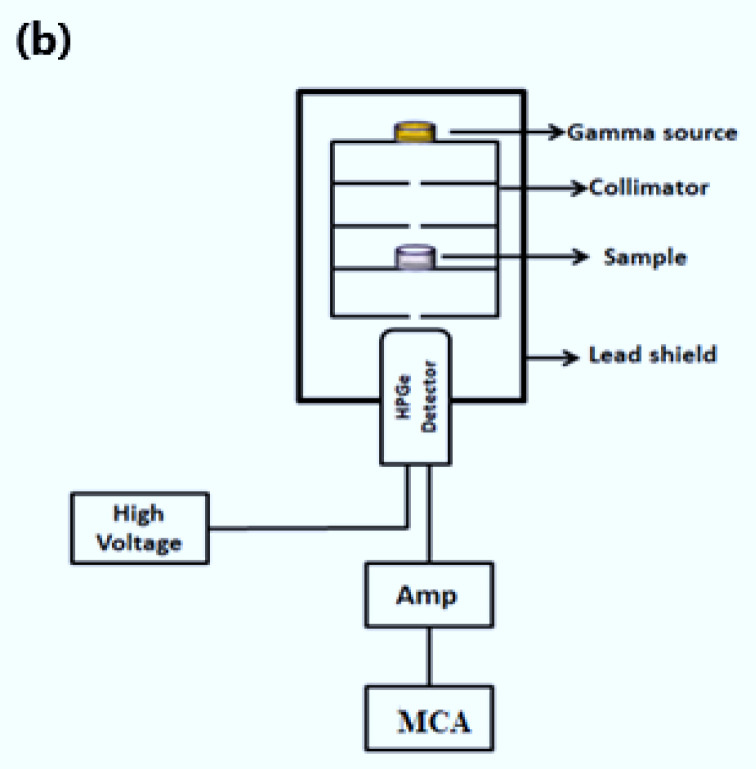
(**a**) HPGe detector accompanied by lead castles and electronic systems. (**b**) Schematic diagram of the experiment setup.

The experiments have been carried out using a cylindrical detector with p-type Hyper Pure Germanium (HPGe) (Model GC1520, Canberra, Australia) with a relative efficiency of 15% in the energy range from 50 keV to 10 MeV [23,24]. Through the narrow beam geometry, the gamma radiation transmittance can be calculated. We used the experimental setup shown in Figure 3, where the detector was surrounded and cylindered by a lead shield with a thickness of 40 mm and a height of 135 mm to minimize background radiation. The distance between the radioactive source and the detector’s surface is 112 mm, ensuring an acceptable narrow beam geometry. Canberra’s ISO 9001 Genie 2000 data acquisition and analysis software, (9233652E V3.0) was used to record and process the measurements, performed using a multichannel analyzer (MCA) to acquire statistically significant peaks in the spectra. The acquisition time was high enough to make the statistical uncertainties less than 1%. Then, the gamma analyzer software automatically calculated the peak area [25].

For each sample, the experimental measures are performed twice. Without taking a sample, a radiation count reading is first collected to determine the source’s intensity (I_o_). The sample is included in the measurement, which shows the transmitted gamma radiation intensity (I). Based on these values I_o_ and I, a linear attenuation coefficient (LAC) is calculated using the Beer–Lambert law, as shown in the following equation [26].

(1) $$\mu =\frac{-1}{x}\;\ln\frac{I}{I_{o}}$$

where μ is the linear attenuation coefficient (LAC) and x is the thickness of the sample. The half value layer (HVL) is defined as the required absorber thickness, which decreases the intensity of the incident beam of photons to half its initial value after passing through the material of the absorber, whereas the tenth value layer (TVL) is the absorber thickness, which reduces the incident photon intensity to a tenth of its initial value after crossing through the material of the absorber [27]. They are calculated by

(2) $$\mathrm{HVL}=\frac{\mathrm{LN}\;\left(2\right)}{\mu }$$

(3) $$\mathrm{TVL}=\frac{\mathrm{LN}\;\left(10\right)}{\mu }$$

The mean free path (MFP) represents the average distance between two successive interactions between gamma rays and a sample.

(4) $$\lambda =\frac{1}{\mu }$$

Furthermore, the mass attenuation coefficient (MAC) can be calculated using the following equation, where (*ρ*) is the density of the sample:

(5) $$\mathrm{MAC}=\frac{\mu }{\rho }$$

The effective atomic number (Z_eff_) of the composites is given by the relation.

(6) $$Z_{\mathrm{eff}}=\frac{\Sigma_{i\;}w_{i}\;A_{i}\;{\left[\frac{\mu }{\rho }\right]}_{i}}{\Sigma_{i\;}w_{i\;}\frac{A_{i}}{z_{i}}\;{\left[\frac{\mu }{\rho }\right]}_{i}}$$

where Z_i_, A_i_, and w_i_ are atomic number, atomic weight, and the weight fraction of element i in composite, respectively.

The effective electron density (N_eff_) is derived from the effective atomic number and is defined as the number of electrons per unit mass.

(7) $$N_{\mathrm{eff}}=\frac{Z_{\mathrm{eff}}{\;N}_{A}}{\sum_{i}w_{i}A_{i}}$$

where N_A_ and A_i_ are Avogadro’s number and the molar mass of a related element in the mixture, respectively [28]. 

The exposure buildup factor (EBF) is a valuable parameter for assessing multiple scattering in radiation shield design. Using the G-P fitting method, the EBF of the samples was calculated [29,30,31], which is supposed to be obtained by the equivalent atomic number (Z_eq_), which is a parameter that describes the properties of mortar samples in terms of equivalent elements. As the accumulation of photons predominantly results from multiple scattering, which is primarily caused by the Compton scattering process, it is important to note that this number is allocated to composite materials by yielding a heavy weight to this process. As a result, the Compton partial mass attenuation coefficient forms the basis for the calculation of Z_eq_, as shown in the following equation [32,33,34].

(8) $$z_{\mathrm{eq}}=\frac{z_{1}(\log R_{2}-\log R)+z_{2}(\log R-\log R_{1\;})}{\left(\log R_{2}-\log R_{1}\right)}$$

where R is the ratio (µ_Comp_/µ_total_) that lies between R_1_ and R_2_ for the selected mortars within a specific energy range. R_1_ and R_2_ are (μ_Comp_/μ_total_) ratios corresponding to elements that have the atomic numbers Z_1_ and Z_2_, respectively. Using the Z_eq_ values for the selected mortars, the G-P fitting parameters (b, a, X_k_, x, and c) in the energy range of 0.015–15 MeV using the following interpolation equation [32,33,34]:

(9) $$C=\frac{C_{1}(\log z_{2}-\log z_{\mathrm{eq}})+C_{2}(\log z_{\mathrm{eq}}-\log z_{1})}{\left(\log z_{2}-\log z_{1}\right)}$$

where C_1_ and C_2_ are the values of the G-P fitting coefficients corresponding to the elements with atomic numbers Z_1_ and Z_2_, respectively. Finally, the EBF for the selected mortar samples is then estimated at a specific energy and up to 40 mfp penetration depth using the following relations [32,33,34]:

(10) $$B\left(E,x\right)=1+\frac{b-1}{K-1}\left(K^{x}-1\right)\;,\;K\neq 1$$

(11) $$B\left(E,x\right)=1+\left(b-1\right)\;x\;,\;K=1$$

where

(12) $$K\left(E,x\right)={\mathrm{cx}}^{a}+d\frac{\tanh\left(\frac{x}{X_{k}}-2\right)-\tan h\left(-2\right)}{1-\tan h\left(-2\right)}\;\mathrm{for}\;x\;\leq 40\;\mathrm{mfp}$$

## 3. Results and Discussion

To verify the precision of the experimental results, the experimental values of the mass attenuation coefficient (MAC) of the mortar samples (CS, CS-mBi_2_O_3_ 15%, and CS-mBi_2_O_3_ 30%) were compared with the theoretical values using the XCOM [35] program based on the chemical composition of each sample. Table 4 displays the experimental and XCOM values of the MAC, as well as the deviation between these two methods calculated by Equation (13).

(13) $$\Delta \;\%=\left(\frac{{\mathrm{MAC}}_{\mathrm{XCOM}}-{\mathrm{MAC}}_{\mathrm{EXP}}}{{\mathrm{MAC}}_{\mathrm{EXP}}}\right)\times 100$$

Both methods are in perfect agreement with each other, where the values are varied between 0.03% and 3.96%. The sample composition and the photon energy are two significant factors that influence the mass attenuation coefficients (MAC). Table 4 shows how the percentage of Bi_2_O_3_ increases MAC values. To be more specific, the MAC of CS < CS-mBi_2_O_3_ 15% < CS-mBi_2_O_3_ 30%. For example, at 0.0595 MeV energy, the MAC values for CS, CS-m Bi_2_O_3_ 15%, and CS-m Bi_2_O_3_ 30% are 0.2825 ± 0.0031, 0.8005 ± 0.0018, and 1.3128 ± 0.0024 cm^2^/g, respectively. Additionally, Table 4 displays the experimental values of the mass attenuation coefficient (MAC) of CS-nBi_2_O_3_ 15%, and CS-nBi_2_O_3_ 30% samples. When calculating the relative increase factor which is defined by Equation (14), it is clear that the MAC of the mortar with nano Bi_2_O_3_ is higher than that of micro Bi_2_O_3_ at all energies.

(14) $$R.I\;\%=\left(\frac{{\mathrm{MAC}}_{\mathrm{nano}}-{\mathrm{MAC}}_{\mathrm{micro}}}{{\mathrm{MAC}}_{\mathrm{micro}}}\right)\times 100$$

Furthermore, Table 4 shows that increasing the gamma-ray energy resulted in a decrease in the mass attenuation coefficients (MAC). However, the presence of the Bi K-edge (90.52 keV) causes a jump in MAC at energy 0.1218 MeV for all samples.

**Figure 4:**
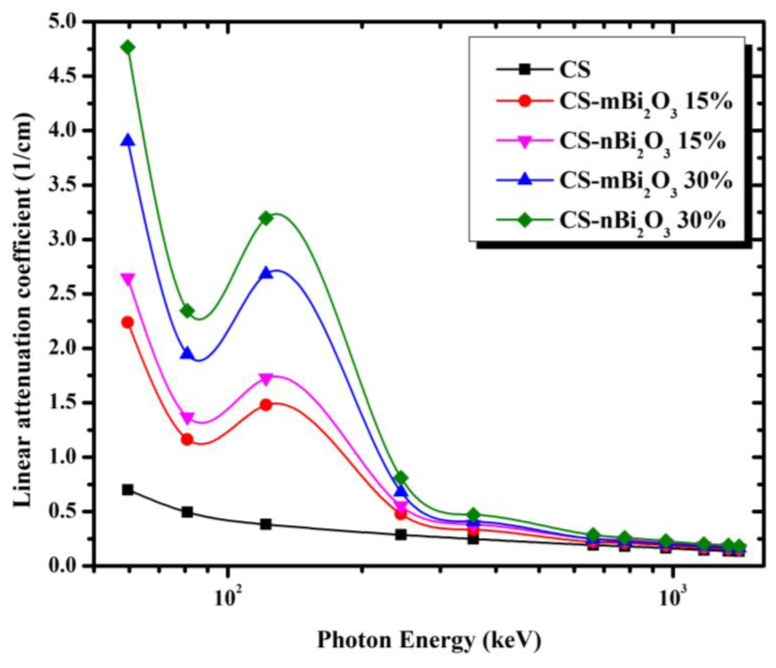
The linear attenuation coefficient of all mortar samples as a function of the photon energy (MeV).

Figure 4 shows the LAC of the prepared mortars with varying amounts of micro and nano Bi_2_O_3_. We can see that the relationship between LAC and energy is inversely proportional, where when the energy is low, the LAC values are high, and vice versa. However, at energy 0.1218 MeV there is a sudden increase in LAC, and this increase is attributed to the presence of the K-edge of Bi (90.52 keV). As a result, it may be concluded that all prepared samples have a high ability to attenuate low energy radiation, especially at the K-edge value, and that this ability gradually declines as photon energy increases. Additionally, we found that the LAC of mortar with nano Bi_2_O_3_ is higher than that of micro Bi_2_O_3_ at all energies and that is because mortar with nano Bi_2_O_3_ contains more Bi_2_O_3_ particles per gram than mortar with micro Bi_2_O_3_. Therefore, there is a consistent distribution and diffusion inside the mortar. As a result, mortar containing nano Bi_2_O_3_ may have higher photon interaction and absorption rates than mortar containing micro Bi_2_O_3_.

**Figure 5:**
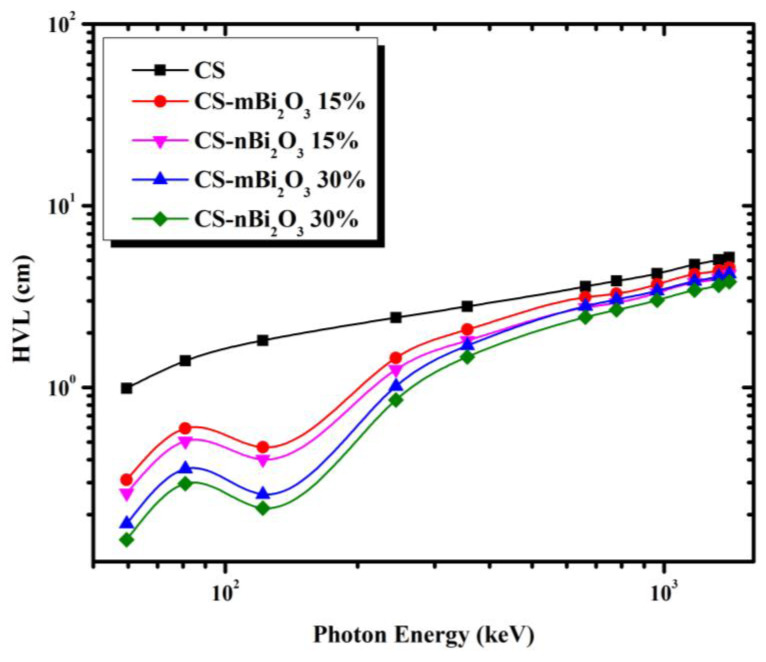
Half value layer (HVL) for the prepared mortars as a function of photon energy.

**Figure 6:**
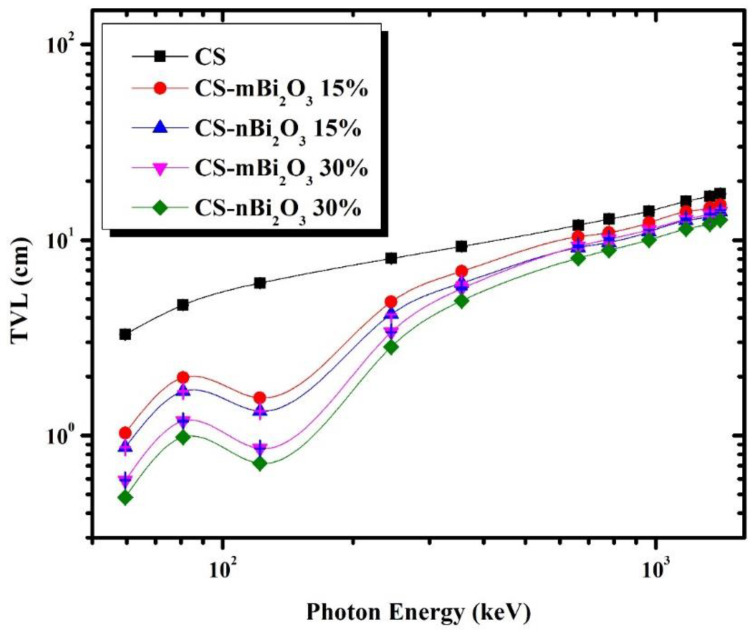
Tenth value layer (TVL) for the prepared mortars as a function of photon energy.

**Figure 7:**
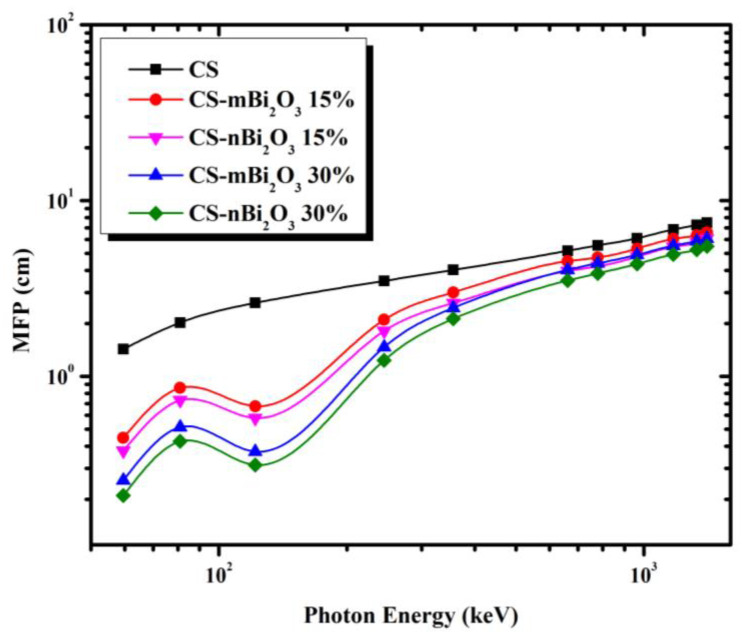
Variation of the mean free path (MFP) with the photon energy.

For discussing the shielding capabilities of prepared mortar for gamma-rays, some parameters based on LAC were calculated, such as half value layer (HVL), tenth value layer (TVL), and mean free path (MFP); the lower they are, the better. Figure 5, Figure 6 and Figure 7 display the variation in HVL, TVL, and MFP values among all samples as a function of incident photon energy and show that these parameters are photon energy dependent where increasing incident photon energy results in an increasing of HVL and TVL as well as MFP values. This means that, as the energy of the photon increases, the probability of a photon interacting with the sample is reduced, allowing for greater photon penetration. However, the values of these parameters in Bi_2_O_3_ doped mortars decrease at energy 0.1218 MeV due to the presence of the Bi K-edge (90.52 keV). Furthermore, Figure 5, Figure 6 and Figure 7 show that CS has the highest HVL, TVL, and MFP at all energies, while the mortar sample with 30% Bi_2_O_3_ nano has the lowest, indicating that the former is less efficient while the latter is more efficient.

**Figure 8:**
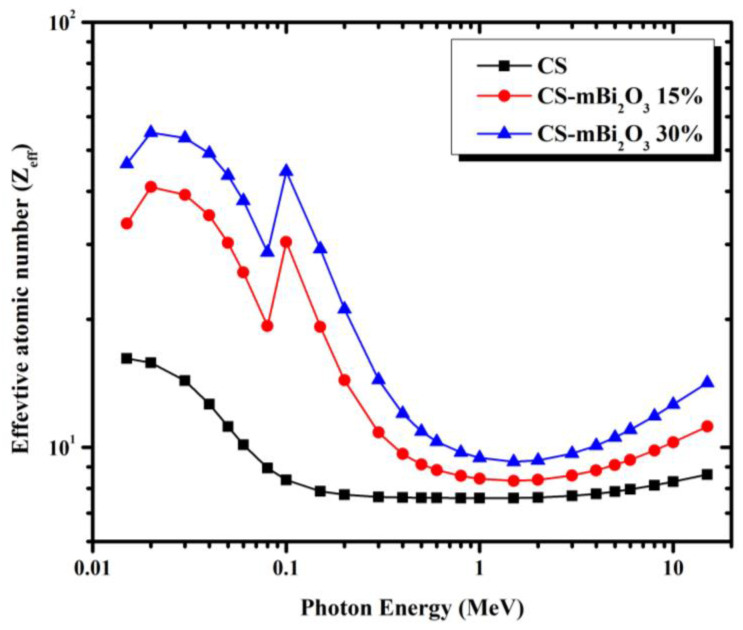
Variation of the effective atomic number (Z_eff_) of mortar samples as a function of energy.

**Figure 9:**
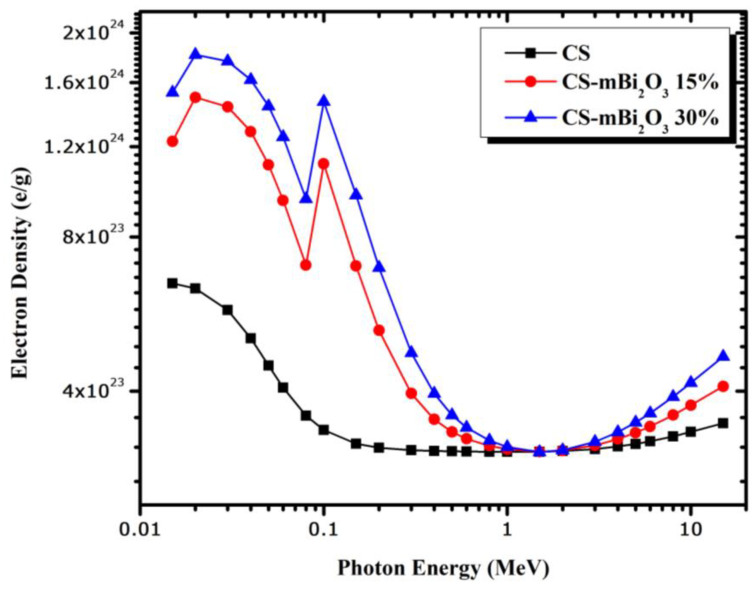
The variation of electron density (N_eff_) of mortars as a function of energy.

The effective atomic number (Z_eff_) was also established to appreciate the efficacy of shielding material attenuation capabilities for the prepared mortars. The probability of photoelectric absorption increases with a high effective atomic number. The mortar sample with the highest concentration of Bi_2_O_3_ (CS-mBi_2_O_3_ 30%) showed a steadily increasing linear attenuation coefficient of gamma rays, as shown in Figure 4, due to its association with high Z_eff_ values. Figure 8 depicts the calculated effective atomic number (Z_eff_) of mortar samples from 0.015 to 15 MeV. Initially, Z_eff_ has comparatively high values and maximum values occur at 0.1 MeV for the mortars with Bi_2_O_3_ contents., because of the K-absorption of Bi. As energy increases up to 1 MeV, Z_eff_ drops dramatically to minimum values, indicating that the Compton scattering process takes over. Due to the dominance of the pair creation process in the high energy region, the values of Z_eff_ increased slowly with energy. Figure 9 shows the electron density (N_eff_) curves for the mortar samples, which exhibit behavior that is quite similar to that of the Z_eff_ curves.

**Figure 10:**
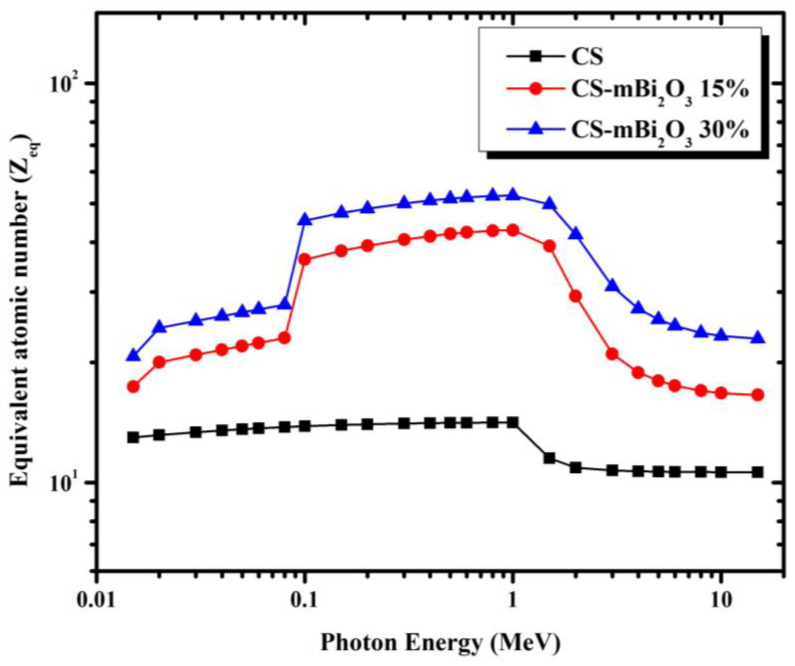
The variation of the Z_eq_ of mortars as function incident gamma ray energy.

The equivalent atomic number (Z_eq_) is a parameter that is utilized in the computation of the exposure buildup factor (EBF) and it describes the shielding characteristics of mortar samples in terms of equivalent element. Figure 10 depicts the calculated equivalent atomic number (Z_eq_) of mortar samples for an energy range of 0.015 to 15 MeV. It is observed that the values of Z_eq_ increase in the middle energy region in the range (0.1 MeV < E < 1 MeV), due to the Compton effect process, but decrease in the low and high energies. Additionally, Figure 10 shows the direct relationship between the Bi content in the samples and Z_eq_, where the sample CS-mBi_2_O_3_ 30% has the highest value of Z_eq_, while the sample CS has the lowest value.

**Figure 11:**
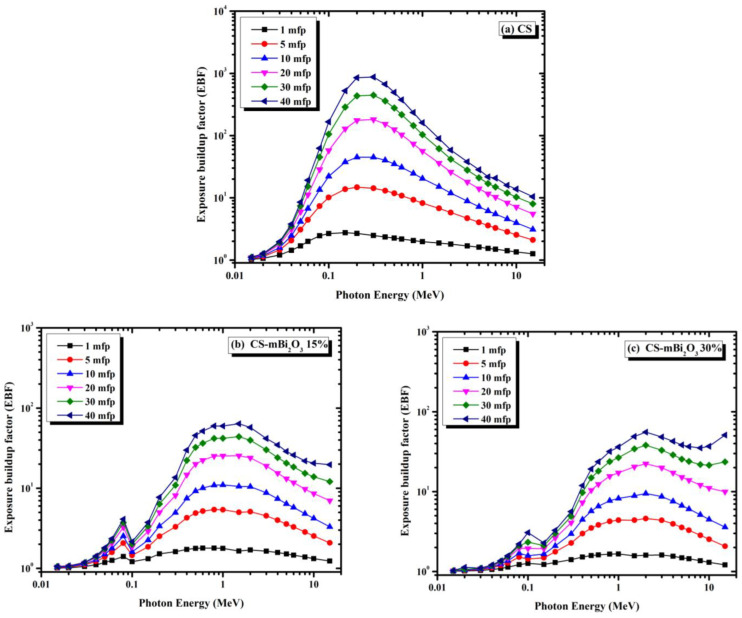
The variation of exposure buildup factor (EBF) with gamma ray energy for different samples.

Figure 11a–c shows how the exposure build-up factor (EBF) varies with photon energy for penetration depths of up to 40 mfp for the samples CS, CS-mBi_2_O_3_, and CS-mBi_2_O_3_. EBF typically increases as the penetrating depth increases; however, the impact was variable and was reported to be energy dependent. The abundance of the photoelectric effect is represented by the lowest EBF values at low energy levels (0.1 MeV), and the K-absorption edge of Bi is attributed to a sharp peak at 0.09 MeV (this peak was observed in samples of CS-m Bi_2_O_3_ 15% and CS-m Bi_2_O_3_ 30%). In the intermediate energy range (0.1 to 10 MeV), EBF rises with energy and reaches its maximum value at 1.5 MeV, which is predicted due to the likelihood of the Compton scattering effect. At very high energies (more than 10 MeV), due to the pair production event, the values tended to steadily rise as incident energy increased. The probability of pair production increases with photon energy and atomic number. Consequently, numerous scattering events cause a buildup of secondary gamma photons produced by electron-positron annihilation in the medium. Additionally, the increased thickness of the interacting material as a result of the increased penetration depth of the materials causes an increase in scattering events in the interacting medium, particularly for the material with the greatest equivalent atomic number. As a result, the EBF values are high [36].

## 4. Conclusions

This work evaluated the radiation shielding properties and buildup factor of five mortar mixture groups, which are coded as CS, CS-mBi_2_O_3_ 15%, CS-mBi_2_O_3_ 30%, CS-nBi_2_O_3_ 15%, and CS-nBi_2_O_3_ 30%. The mass attenuation coefficient (MAC) was determined experimentally, the experimental results were validated by XCOM software [35], and the results agreed. The attenuation coefficients of the composites were found to increase dramatically as the content of the Bi_2_O_3_ fillers in the mortar increased. Furthermore, due to the high surface-to-volume ratio of the Bi_2_O_3_ nanoparticles compared to the micro composites, the attenuation coefficients of the nanocomposites were noticeably higher than those of the micro composites. In addition, we investigated the gamma radiation parameters HVL, TVL, and MFP, which showed the superiority of Bi_2_O_3_ nanoparticles, which recorded the lowest value compared to Bi_2_O_3_ microparticles, especially at a concentration of 30%. The morphological test was also carried out using SEM, where the nanoparticles improved the morphological properties. We conclude that Bi_2_O_3_ nanoparticles can effectively serve as a gamma-ray shield and, because of their low toxicity, they can be added to building materials and used in nuclear and medical facilities. 

## Figures and Tables

**Table 1:** Mineral chemical composition as weight fraction in percentage (w%).

Element	White Cement (w%)	Sand (w%)
C	0.0927	0.136
O	0.5080	0.561
Al	0.0105	0.0034
Si	0.0660	0.0042
S	0.0228	0.0008
Ca	0.3000	0.285
Na	-	0.0015
Mg	-	0.0027
K	-	0.0024
Ti	-	0.0011
Fe	-	0.0019

**Table 2:** Mixing mass ratio of mortar samples.

Sample	Density g/cm^3^	Cement	Fine Sand	Bi_2_O_3_ Microparticles	Bi_2_O_3_ Nanoparticles
Pure CS	1.477 ± 0.002	1	3	0	0
CS-mBi_2_O_3_ 15%	2.795 ± 0.005	1	2.7	0.3	-
CS-m Bi_2_O_3_ 30%	2.972 ± 0.003	1	2.1	0.9	-
CS-n Bi_2_O_3_ 15%	2.786 ± 0.004	1	2.7	-	0.3
CS-n Bi_2_O_3_ 30%	3.439 ±0.003	1	2.1	-	0.9

**Table 3:** PTB point source activities and their uncertainties.

PTB	Nuclide Activity (kBq)	Reference Date	Uncertainty (kBq)
^241^Am	259.0	00:00 Hr 1. June 2009	±2.6
^133^Ba	275.3		±2.8
^152^Eu	290.0		±4.0
^137^Cs	385.0		±4.0
^60^Co	212.1		±1.5

**Table 4:** Experimental and theoretical values of MAC of all samples at different photon energies.

Sample	Energy (MeV)	Mass Attenuation Coefficient (cm^2^ g^−1^)				
		Micro	XCOM	∆ %	Nano	R.I %
CS	0.0595	0.2825 ± 0.0031	0.2838	−0.47		
	0.0810	0.1994 ± 0.0011	0.1999	−0.26		
	0.1218	0.1540 ± 0.0024	0.1537	0.18		
	0.2447	0.1154 ± 0.0022	0.1166	−1.05		
	0.3560	0.1001 ± 0.0009	0.1011	−1.03		
	0.6617	0.0779 ± 0.0028	0.0781	−0.24		
	0.7789	0.0725 ± 0.0032	0.0725	0.03		
	0.9641	0.0661 ± 0.0033	0.0655	0.85		
	1.1730	0.0588 ± 0.0005	0.0595	−1.08		
	1.3320	0.0555 ± 0.0005	0.0558	−0.53		
	1.4080	0.0538 ± 0.0002	0.0542	−0.72		
CS-m Bi_2_O_3_ 15%	0.0595	0.8005 ± 0.0018	0.8255	−3.03	0.9498 ± 0.0062	18.65%
	0.0810	0.4160 ± 0.0036	0.4308	−3.45	0.4909 ± 0.0041	18.01%
	0.1218	0.5291 ± 0.0004	0.5350	−1.10	0.6195 ± 0.0013	17.09%
	0.2447	0.1705 ± 0.0021	0.1749	−2.52	0.1981 ± 0.0040	16.17%
	0.3560	0.1191 ± 0.0031	0.1219	−2.33	0.1372 ± 0.0041	15.27%
	0.6617	0.0791 ± 0.0030	0.0815	−2.92	0.0903 ± 0.0085	14.12%
	0.7789	0.0755 ± 0.0012	0.0745	1.34	0.0850 ± 0.0026	12.64%
	0.9641	0.0671 ± 0.0002	0.0663	1.18	0.0749 ± 0.0062	11.66%
	1.1730	0.0591 ± 0.0032	0.0597	−1.02	0.0657 ± 0.0051	11.20%
	1.3320	0.0563 ± 0.0051	0.0558	1.07	0.0622 ± 0.0033	10.45%
	1.4080	0.0543 ± 0.0005	0.0542	0.33	0.0591 ± 0.0016	8.78%
CS-m Bi_2_O_3_ 30%	0.0595	1.3128 ± 0.0024	1.3670	−3.96	1.6061 ± 0.0044	22.34%
	0.0810	0.6543 ± 0.0031	0.6618	−1.13	0.7846 ± 0.0021	19.91%
	0.1218	0.9026 ± 0.0008	0.9165	−1.52	1.0655 ± 0.0039	18.05%
	0.2447	0.2293 ± 0.0005	0.2332	−1.65	0.2704 ± 0.0026	17.92%
	0.3560	0.1372 ± 0.0034	0.1427	−3.88	0.1596 ± 0.0015	16.39%
	0.6617	0.0833 ± 0.0029	0.0849	−1.86	0.0960 ± 0.0034	15.16%
	0.7789	0.0765 ± 0.0035	0.0764	0.11	0.0871 ± 0.0082	13.88%
	0.9641	0.0685 ± 0.0001	0.0671	2.09	0.0768 ± 0.0033	12.02%
	1.1730	0.0607 ± 0.0022	0.0599	1.33	0.0678 ± 0.0003	11.70%
	1.3320	0.0572 ± 0.0009	0.0558	2.51	0.0634 ± 0.0063	10.91%
	1.4080	0.0553 ± 0.0027	0.0541	2.12	0.0605 ± 0.0094	9.43%

## Data Availability

All data are available in the manuscript.
